# The association between the COVID-19 pandemic and interpersonal relationships among youth with a child maltreatment history

**DOI:** 10.3389/frcha.2024.1434496

**Published:** 2024-11-29

**Authors:** Julie-Anne McCarthy, Ana M. Osorio, Tamara L. Taillieu, Ashley Stewart-Tufescu, Tracie O. Afifi

**Affiliations:** ^1^ Department of Community Health Sciences, Max Rady College of Medicine, University of Manitoba, Winnipeg, MB, Canada; ^2^ Faculty of Social Work, University of Manitoba, Winnipeg, MB, Canada; ^3^ Department of Psychiatry, Max Rady College of Medicine, University of Manitoba, Winnipeg, MB, Canada

**Keywords:** child maltreatment (CM), adverse childhood experiences (ACES), COVID-19 pandemic, conflict, parent-youth relationships, sibling relationships, intimate partner relationships, youth & emerging adult well-being

## Abstract

**Introduction:**

The COVID-19 pandemic had significant impacts on youth health and well-being. Youth with prior inequities, such as those exposed to child maltreatment, may have experienced greater psychosocial challenges and long-term difficulties than their peers, including sustained interpersonal relationships problems. Given the importance of healthy relationships during adolescence and early adulthood, the significant impact the pandemic had on youth, and the potential disproportionate challenges for youth with a child maltreatment history, the purpose of the present study was to better understand changes in relational conflict among youth with and without a child maltreatment history from the perspectives of youth themselves. Specifically, the aims were to examine if youth child maltreatment history was associated with an increased likelihood of reporting increased conflict with (a) parents, (b) siblings, or (c) intimate partners during the first three years of the COVID-19 pandemic.

**Methods:**

Data were drawn from the Well-Being and Experiences (WE) Study; a longitudinal and intergenerational cohort study of 1,000 youth/parent dyads in Manitoba, Canada that began in 2017. WE study data were collected annually via self-reported youth surveys between 2017 and 2022 for a total of 5 waves of data collection, and COVID-19 questions were included in Waves 3 (2020), 4 (2021) and 5 (2022) (*n* = 586, 56.43% female, ages 18 to 21 at Wave 5). Multinomial regressions models were computed to examine whether a youth's child maltreatment history was associated with increased, decreased, or consistent levels of conflict with parents, siblings, and intimate partners in 2020, 2021, and 2022 compared to pre-pandemic levels.

**Results:**

Results indicated that compared to youth with no child maltreatment history, youth with a child maltreatment history were more likely to report increased conflict across all three types of relationships during first three years of the pandemic.

**Discussion:**

Findings contribute to our understanding of the association between the COVID-19 pandemic and interpersonal relationships among youth who have a child maltreatment history compared to their peers without child maltreatment histories, signal potential long-term challenges or inequities for youth and families with a history of maltreatment, and may inform policy, programming, intervention, and recovery efforts in the post-COVID-19 period, and for future global emergencies.

## Introduction

Disruptions caused by the COVID-19 pandemic impacted youth globally (1, 2). Several studies reported increased mental health challenges, academic difficulties, employment and financial issues, and isolation for young people around the world (3–7). Certain key populations were at an even higher risk of experiencing difficulties, inequities or harm during the pandemic; among these were youth with child maltreatment experiences (6, 8–11). Child maltreatment is defined as any abuse or neglect that occurs before the age of 18 years, and includes physical, sexual, and emotional abuse, physical neglect, emotional neglect, and exposure to intimate partner violence (EIPV) (12, 13). It is a prevalent and harmful public health issue that affects health and well-being throughout the life course (14–19). Physical health challenges include chronic headaches and migraines, back pain, fatigue, high blood pressure, obesity, stroke, respiratory issues, bowel disease, and cancer (20, 21). Psychological challenges include anxiety and depression, post-traumatic stress disorders, risk-taking behaviours, substance use problems, poorer quality of life, lower self-perceived health and well-being, and suicide (16, 22–27). Furthermore, children and youth who experience maltreatment are frequently exposed to multiple forms of adverse childhood experiences (ACEs) (e.g., abuse, poverty, parental mental health challenges, school bullying) (23, 28–32), and the cumulative effects of multiple maltreatment and adversity exposures pose a great risk on child and youth outcomes, particularly given the critical developmental period during which these occur (16, 17, 26, 27, 31, 33).

The COVID-19 pandemic exacerbated difficulties for youth with child maltreatment histories. Prior to the COVID-19 pandemic, international past-year prevalence studies estimated that one billion children between 2 and 17 years old experienced physical, sexual, and emotional violence from adults and peers (34, 35). In Canada, pre-pandemic studies indicated that 32% of adults reported experiences of physical abuse, sexual abuse and/or EIPV during childhood or adolescence, 26.1% reported physical abuse experiences, 10.1% reported sexual abuse experiences, 7.9% reported EIPV (27), and 59.7% reported any type of child maltreatment experience before the age of 16 years old (36). Following the onset of COVID-19, the Center for Disease Control and Prevention (CDC) estimated that 11% of youth experienced physical abuse and 55% experienced emotional abuse by a parent within the first year of the pandemic (10, 37). Violence against women (38), intimate partner violence (8, 39, 40), and online dating violence among teens also increased during the pandemic (40, 41). Sibling relationships may also have been affected, although the literature is limited and mixed, with some reporting increased closeness and others reporting increased tension (42–45). While some studies assessing family dynamics during pandemic “lockdown” periods indicated increased family cohesion (46, 47), for young people who were already experiencing abuse, neglect, or violence in their home, closer contact with the abuser, increased isolation, limited contact with social networks, and reduced detection or reporting of abuse may have risked worsening dangerous and vulnerable situations (5, 10, 37, 48–50). Parental job loss, financial challenges or poverty, overcrowding, and concerns of disease-related death or illness heightened parental distress and may also have been contributing factors (6, 8, 9, 37, 46, 48, 49, 51). Examining differences of the impact of COVID-19 among youth with and without maltreatment histories is needed to better understand potential discrepancies in experiences and long-term implications.

Interpersonal relationships are critical components of youth health and development that can either act as protective factors that buffer against stressors and mitigate other negative outcomes (40, 46, 47, 52), or as risk factors that further perpetuate negative outcomes (45, 52–54). Pre-pandemic studies established that child maltreatment increases the risk of interpersonal difficulties in adolescence and adulthood (53, 55), including dating violence victimization or perpetration (40, 52), poorer parent-child relationships, and future parenting practices (56). To date, pandemic-based studies have indicated that maltreatment increased the odds of experiencing educational and financial difficulties (7), alcohol and cannabis use (6), and anxious or depressed feelings (5, 57). Child maltreatment also may have had a moderating role between pandemic-related distress and well-being outcomes, including more internalized and externalized behaviours, and lower self-esteem and life satisfaction for youth with a child maltreatment history (11). In addition, youth who experienced negative impacts of COVID-19 on family and peer relationships were more likely to report lower life satisfaction and higher physical and psychological health complaints than youth who reported a neutral impact from the pandemic (58). However, less is known about the immediate and intermediate impacts of COVID-19 on relational conflict from the perspectives of youth with maltreatment histories in particular; many maltreatment studies to date have focused on younger children rather than adolescents or emerging adults (46, 47), have included proxies (e.g., parents or teachers) rather than youth, and have been retrospective and cross-sectional—which are subject to recall bias (5)—and did not specifically consider pre-existing maltreatment experiences on relationship outcomes.

The World Health Organization (WHO) and the United Nations (UN) have projected that abuse prevalence may remain at higher than pre-pandemic levels (8, 10), which risks to further widen the gap between youth with a child maltreatment history and their peers. As such, longitudinal research capturing the impact of the pandemic on relationships from the perspectives of youth themselves is critical to identify patterns, understand potential long-term issues, and inform areas for policy, programs and interventions that are founded on youth voices (1, 5, 49, 59, 60). The purpose of the present study was to examine the association between the COVID-19 pandemic and relational conflict among youth with a child maltreatment history using longitudinal self-reported youth surveys. Specific aims were to examine whether youth with a child maltreatment history (i.e., abuse, neglect, and EIPV), had a higher risk of reporting increased conflict with parents, siblings, and intimate partners during the first three years of the COVID-19 pandemic (2020, 2021, 2022) compared to youth without a child maltreatment history. It was hypothesized that youth with a child maltreatment history would be at greater risk of reporting increased relational conflict during the pandemic across all types of relationships compared to their peers without a child maltreatment history.

## Materials and methods

### Data and sample

**Figure 1:**
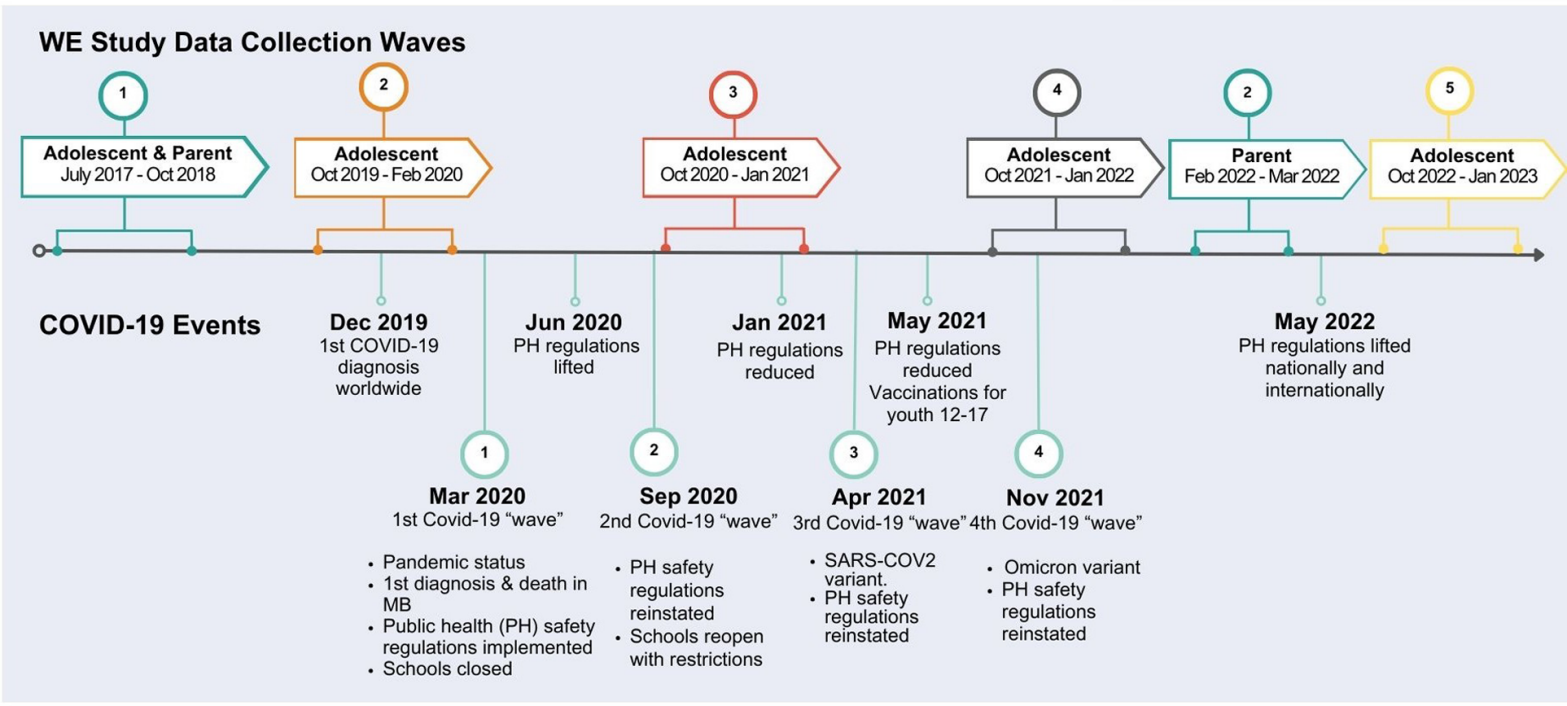
Timeline: well-being and experiences (WE) study data collection and COVID-19 events in Manitoba, Canada.

Data were drawn from the Well-being and Experiences (WE) study, a multi-wave community-based longitudinal, intergenerational cohort study of 1,000 youth-parent/caregiver dyads that began in 2017. Participants were recruited through random digit dialing and convenience sampling in Manitoba, Canada (15). At baseline (2017), youth participants were 14 to 17 years old (mean age = 15.3 years; 51.7% female), and closely approximated the population that lived in urban and rural areas of the province (i.e., Winnipeg and surrounding areas) based on sex, ethnicity, and household income. Youth completed these self-reported questionnaires annually between 2017 and 2022 for a total of 5 waves of data collection (Figure 1). Questionnaires were completed in person for Wave 1, then remotely for Waves 2 to 5. The WE study questionnaires included several questions on multiple aspects of physical and mental health, school, family and employment, intimate partner relationships, child maltreatment history, resilience, and protective factors. COVID-19 questions were included in Waves 3 (2020), 4 (2021) and 5 (2022). Due to reporting laws among minors, child maltreatment questions (childhood physical, sexual, and emotional abuse, physical neglect, and emotional neglect) were included in surveys for youth 18 years and older (7, 61, 62). Responses from youth 18 years and older at Waves 3, 4, and 5 (*n* = 586) were therefore included in the present study analyses to examine COVID-specific responses. Participation in the study was voluntary; adolescents and parents provided informed consent and were aware that they could withdraw at any time. Individuals who participated in a survey were contacted via the email address that they had provided to participate in the next round of data collection. If they indicated that they were interested in participating, a link was emailed to them and the informed consent appeared when they first clicked on the link. If they provided informed consent, they proceeded to the online questionnaire, if they did not provide consent, the survey ended, and they were not contacted for future data collection waves. Ethics approval was provided from the University of Manitoba Health Research Ethics Board (15).

### Measurement

#### Child maltreatment

Child maltreatment was assessed using the validated and reliable 28 item Childhood Trauma Questionnaire (CTQ) (61, 62). The CTQ is designed to assess childhood physical, sexual, and emotional abuse, physical neglect, and emotional neglect, each of which are measured with five questions on a 5-point ordinal scale (never true to very often true) (61, 62). EIPV was assessed using the Childhood Experiences of Violence Questionnaire (CEVQ) which asks participants to report how many times they saw or heard any one of their parents, step-parents or guardians hit each other or another adult in their home before the age of 16 (63); response options included *(1) Never, (2) 1 or 2 times, (3) 3 to 5 times, (4) 6 to 10 times, (5) 11 to 20 times, (6) More than 20 times, (7) I don't know,* or (*8) No response.* Responses to the CTQ and CEVQ were coded as binary (yes/no) with different cut-off scores for each type of abuse (physical abuse cut-off score of eight or above; sexual abuse, six or above; emotional abuse, 13 or above; emotional neglect, 15 or above; physical neglect, eight or above; and EIPV, three or above); cut-off scores were determined based on guidelines provided by the original tool developers Bernstein et al. (61) and used in previous studies (61, 62, 64). Each type of abuse was then added together to create an overall child maltreatment composite variable of “yes” (exposure to one or more types of child maltreatment) vs. “no” (no child maltreatment exposure) (Table 1).

**Table 1:** Sociodemographic characteristics and child maltreatment responses among youth 18 to 21 years old at Waves 3, 4, and 5 of data collection.

Sociodemographic characteristics	% (*n*)
Sex (*n* = 583)	
Male	43.57 (254)
Female	56.43 (329)
Household income (*n* = 560)	
$49,999 or less	15.89 (89)
$50,000 to $99,999	38.57 (216)
$100,000 to $149,999	23.04 (129)
$150,000 or more	22.50 (126)
Ethnicity (*n* = 576)	
White only	62.85 (362)
White and another	20.31 (117)
Other/multi-ethnicity	16.84 (97)
Child Maltreatment (*n* = 586)	
Yes	55.0 (322)
No	45.0 (264)

#### Conflict with parents, siblings, and intimate partners during COVID-19

In the interest of time and in order to capture impacts of the pandemic as it progressed, the following three questions were developed by the research team and added to the WE questionnaires in 2020, 2021, and 2022: *“Has conflict with your parents changed due to the COVID-19 pandemic?”, “Has conflict with your siblings changed due to the COVID-19 pandemic*?”, “*Has conflict with a partner in an intimate relationship changed due to the COVID-19 pandemic?”.* Participant response options included *(1) Increased, (2) Remained the same, (3) Decreased, (4) I don't know, (5) No response.* These three repeated measure questions were used in the study analyses to assess changes in relational conflict with parents, siblings, and intimate partners.

#### Sociodemographic characteristics

Sociodemographic variables measured at baseline (Wave 1) were used as covariates in the adjusted models and included biological sex (male or female), household income and ethnicity. Sex and ethnicity were assessed at baseline on the youth questionnaire. Ethnicity included three categories: White only, white and other, and other/multi-ethnicity. Information on household income was provided by the parent respondent at baseline and included four options: *$49,999 or less, $50,000 to $99,999, $100,000 to $149,999,* and $*150,000 or more***.**

## Analyses

Descriptive analyses were conducted for youth sociodemographic factors including youth sex, ethnicity, household income, and child maltreatment history (Table 1). Multinomial regressions (Table 2) were conducted to examine whether there were associations between youth child maltreatment history (independent variable) and conflict with parents, siblings, intimate partners during the COVID-19 pandemic (dependent variables) in 2020, 2021, and 2022. Child maltreatment responses were dichotomized (no = no child maltreatment; yes = 1 or more types of child maltreatment exposure), and three levels were used to assess change in conflict with parents, siblings, and intimate partner relationships: *increased conflict*, *decreased conflict*, or *remained the same* (reference category). Participants who indicated that they did not have siblings or were not in a relationship at the time of the survey were not included in the analyses for that given Wave of data collection. Responses from youth with a child maltreatment history were compared to those without a child maltreatment history to determine whether responses among these two groups were significantly different from one another. Significance levels of 95% (*p* < 0.05), 99% (*p* < 0.01) and 99.99% (*p* < 0.001) were used to examine whether responses among youth with a child maltreatment history were significantly different from youth with no child maltreatment history. Each wave of data collection was examined independently from one another, in separate models. Regressions were first conducted unadjusted, then adjusted for youth sex, ethnicity, and household income. Study analyses were conducted using STATANow17 software (65).

**Table 2:** Multinomial regression results for conflict during COVID-19 among youth with a child maltreatment history, compared to youth with no child maltreatment history.

	“Increased level of conflict” compared to “remained the same”		“Decreased level of conflict” compared to “remained the same”	
	RR (95% CI)	ARR (95% CI)	RR (95% CI)	ARR (95% CI)
WE Questionnaire response time-periods				
Wave 3 (Oct 2020—Jan 2021)				
Parents	2.10*** (1.33, 3.35)	1.84* (1.14, 2.98)	1.08 (0.60, 1.94)	1.08 (0.58, 2.01)
Siblings	1.74* (1.04, 2.93)	1.63 (0.95, 2.81)	1.46 (0.89, 2.40)	1.34 (0.80, 2.24)
Intimate Partner	2.75** (1.42, 5.35)	2.68** (1.35, 5.31)	1.82 (0.77, 4.28)	1.68 (0.70, 4.03)
Wave 4 (Oct 2021—Jan 2022)				
Parents	2.12*** (1.38, 3.25)	1.85** (1.18, 2.90)	1.65 (0.94, 2.89)	1.38 (0.77, 2.50)
Siblings	1.97* (1.14, 3.39)	1.71 (0.97, 3.02)	1.01 (0.63, 1.62)	0.89 (0.54, 1.47)
Intimate Partner	1.82 (0.96, 3.45)	1.64 (0.84, 3.22)	1.19 (0.51, 2.79)	1.17 (0.48, 2.88)
Wave 5 (Oct 2022—Jan 2023)				
Parents	1.64* (1.00, 2.70)	1.48 (0.88, 2.49)	1.26 (0.69, 2.31)	0.97 (0.51, 1.84)
Siblings	1.25 (0.63, 2.51)	1.12 (0.55, 2.30)	0.98 (0.59, 1.62)	0.81 (0.47, 1.38)
Intimate Partner	2.73* (1.21, 6.16)	2.45* (1.07, 5.63)	1.42 (0.60, 3.33)	1.45 (0.61, 3.42)

RR, risk ratio; ARR, adjusted risk ratio for youth sex, ethnicity, and parent household income; CI, confidence interval.

* *p* < 0.05.

** *p* < 0.01.

*** *p* < 0.001.

## Results

A total of 586 participants 18 to 21 years old (at Wave 5) were included in the analyses. Among them, 56.43% were female, 15.89% had a family income of $49,999 or less, and over half (55%) reported having experienced maltreatment in their childhood or adolescence (Table 1). Multinomial regression models presented in Table 2 indicated whether youth reported changes to levels of conflict throughout the pandemic. In the first year of the pandemic (2020—Wave 3 of the WE study data collection), youth with a child maltreatment history compared to those without a child maltreatment history were significantly more likely to report increased conflict with parents (RR = 2.10; 95% CI 1.33 to 3.35), siblings (RR = 1.74; 95% CI: 1.04 to 2.93) and intimate partners (RR = 2.75; 95% CI 1.42 to 5.35) in the unadjusted model. Increased conflict with parents (ARR = 1.84; 95% CI 1.14 to 2.98) and intimate partners (ARR = 2.68; 95% CI 1.35 to 5.31) remained significant after adjusting for sex, ethnicity and household income, and conflict with siblings was on the margin of significance (ARR = 1.63; 95% CI 0.95 to 2.81). In the second year of the pandemic (2021—Wave 4 of the study), youth with a child maltreatment history compared to youth without a child maltreatment history were more likely to report increased levels of conflict with parents in the unadjusted (RR = 2.12; 95% CI 1.38 to 3.25) and adjusted models (ARR = 1.85; 95% CI 1.18 to 2.90) and with siblings (RR = 1.97; 95% CI 1.14 to 3.39) in the unadjusted model. Conflict with siblings was not significant in the adjusted model and intimate partners was not significant in either unadjusted or adjusted models. In the third year of the pandemic (2022—Wave 5 of the study), youth with a child maltreatment history continued to report significantly more conflict with parents in the unadjusted model (RR = 1.64; 95% CI 1.00 to 2.70) compared to youth without a child maltreatment history; this was no longer significant in the adjusted model. Increased conflict with intimate partners was significant in both the unadjusted (RR = 2.73; 95% CI 1.21 to 6.16) and adjusted models (ARR = 2.45; 95% CI 1.07 to 5.63) at Wave 5. Conflict with siblings was not significantly different from youth without a child maltreatment history in the third year of the pandemic in either unadjusted or adjusted models. Significantly decreased levels of conflict with parents, siblings, or intimate partners were not observed at any point in the data collection during the pandemic.

## Discussion

The COVID-19 pandemic had significant impacts on youth health and well-being (2, 6, 66). Youth with prior inequities, such as those with a child maltreatment history, may have experienced greater psychosocial challenges than their peers (6, 10, 50, 67), and such issues may contribute to long-term difficulties including sustained interpersonal relationships problems with family and intimate partners (1, 52, 56). Given the importance of healthy relationships during adolescence and early adulthood, and the significant disruption of the pandemic on youth lives, the purpose of this study was to better understand changes to levels of relational conflict with parents, siblings, and intimate partners from the perspectives of youth with and without a child maltreatment history.

### Conflict by year of data collection

#### Year 2020

In the first year of the pandemic and following the onset of public health regulations, including lockdowns, school closures, and stay-at-home directives in Manitoba (Figure 1), youth participants with a child maltreatment history were significantly more likely than youth without a child maltreatment history to report increased conflict with their parents, siblings and intimate partners during the pandemic compared to prior to the pandemic. When adjusting for sex, ethnicity and household income, significant results remained for increased conflict with parents and intimate partners, and conflict with siblings was trending towards significance.

#### Year 2021

In 2021, public health regulations changed frequently, shifting from several, to few restrictions numerous times throughout the year. During this time, youth with a child maltreatment history—compared to youth without a child maltreatment history—continued to report significantly more conflict compared to pre-pandemic conflict with their siblings (unadjusted model) and their parents (unadjusted, and adjusted models), but not with intimate partners.

#### Year 2022

By April 2022, most restrictions were lifted in Manitoba; schools, workplaces, healthcare services and extracurricular activities had re-opened, and youth were once again able to socialize in person. At this time, despite an overall “return to normal”, youth with a child maltreatment history were still significantly more likely than youth who did not have a child maltreatment history to report increased conflict with parents (unadjusted) and with intimate partners (unadjusted and adjusted models) when compared to pre-pandemic levels, but not with siblings.

### Conflict by type of relationship

#### Siblings

Results indicated that conflict with siblings was more pronounced at the beginning of the pandemic (2020) but was no longer significantly different for youth with and without a child maltreatment history by the end of the study period (2022). Given that this study included participants in later adolescence and early adulthood, it is possible that these individuals had moved out of their home or away from their siblings by 2022. This may explain some of the reduction in conflict between siblings, however, we do not have information on how living arrangement changed over the pandemic, so this is speculative. There are few studies specifically considering changes to sibling relationship quality during the pandemic, and the results are varied, with some indicating increased closeness during lockdown periods, and others indicating increased tension (42, 43). Sibling relationships are especially important during adolescence as these may be protective against other adversity, or may worsen outcomes depending on the quality of the relationship (42, 43, 45). The present study findings therefore highlight the importance of considering abuse histories within the examination of sibling relationships during and following the onset of the COVID-19 pandemic.

#### Parents

Conflict with parents remained significantly higher than pre-pandemic levels for youth with a child maltreatment history at each wave of data collection (2020, 2021, 2022) in the unadjusted models and was significant in 2020 and 2021 in the adjusted models. Youth who did not have a history of child maltreatment did not report significant increases in conflict with parents at any point of data collection. Adolescent conflict with parents is considered a normative part of individuation at this stage of development, however some COVID-19 studies had indicated potential benefits for family cohesion during the pandemic, such as closer bonds and more quality time spent together (45–47). The fact that the present study results found increased parent-youth conflict only within families with a child maltreatment history is therefore an important finding as this may be indicative of distinct and possibly persistent parent-child relationship problems for these families in particular.

#### Intimate partners

Conflict with intimate partners was significantly higher than pre-pandemic levels in 2020 and 2022 but was not significant in 2021. Participants were not asked to indicate the length of their intimate partner relationships nor whether they were with the same or different partner when participating in each wave of data collection. Study results may therefore reflect changes to intimate partner relationships from the onset of COVID-19 (e.g., youth relationships ending or having less contact after the first lock-down), may indicate momentary reduction of stressors or increased sense of hopefulness (e.g., several local restrictions lifted in 2021, vaccinations became increasingly available), or may be the result of a Type II error due to low statistical power as noted by moderate effect sizes and wider confidence intervals. Other studies examining adolescent dating relationships during COVID-19 had mixed results related to intimate partner relationships, with some reporting increased bonds or closeness, and others reporting increased violence (39–42). In the present study, relationships with intimate partners continued to be reported as more conflictual in 2022 than pre-pandemic levels among youth with a child maltreatment history, and not among those without a child maltreatment history, which may suggest ongoing or increasing issues, either in pre-existing relationships, or new relationships; this is of concern and worth further investigation given the increased rates of overall dating violence and intimate partner violence since the onset of COVID-19 (8, 38–41).

Overall, of prime importance is the fact that youth with a child maltreatment history had significantly different results (i.e., increased conflict) than youth without a child maltreatment history as this identifies disproportionate pandemic-related experiences or inequities for youth who have experienced maltreatment. Furthermore, conflict among youth with a child maltreatment history remained significantly higher in 2022 after most restrictions had been lifted, which suggests potential long-term challenges that may widen gaps in health and psychosocial outcomes and identifies a need for early and ongoing supports. Additionally, youth with a child maltreatment history continued to report increased conflict with intimate partners in 2022, whereas youth without child maltreatment did not report increased conflict with an intimate partner at any point of data collection; this may signify worsening cycles or patterns of conflict or abuse within close intimate relationships as these youth enter adulthood and flags a critical area for intervention.

#### Strengths, limitations, and future directions

Many child maltreatment and pandemic-related studies focused on younger children, rather than youth, included proxies (e.g., parents or teachers) rather than capturing self-reported youth-experiences, and have been cross-sectional (i.e., a single time point) or conducted retrospectively (e.g., after the height of pandemic had passed), which may be subject to recall bias (4, 5, 10, 46, 47). Notable strengths of the current study are the longitudinal study design, conducted directly with youth (rather than proxies), using validated child maltreatment measures, as the COVID-19 pandemic progressed. The assessment of youth's interpersonal relationships at yearly intervals prior-to and during the first three years of COVID-19 reduced the potential of recall bias and captured changes in relational conflict that occurred throughout the pandemic (5). The study design also included youth with and without child maltreatment histories which made it possible to compare responses from these distinct population vantage points, therefore providing valuable information of youth's self-reported experiences throughout this significant point in history. Furthermore, examining changes in relationships with parents, siblings, and intimate partners during adolescence and early adulthood yielded important information about this critical developmental period. The study sample included a cohort who closely represented the youth population in the urban (Winnipeg) and rural areas of Manitoba at baseline. However, due to attrition, the sample used for the analyses may not be representative of the population from which it was drawn. Limited power may have contributed to reduced significance in the adjusted models, possibly resulting in some Type II errors. Although sex and gender differences may exist in interpersonal relationship patterns amongst youth with a child maltreatment history, it was not possible to examine sex or gender differences due to limited power. Furthermore, COVID-19 questions were developed by the research team; this was done in the interest of time, rather than spending time developing a tool, questions were created and added to the surveys to capture the impact of the pandemic as it evolved. Also, although longitudinal, it was not possible to determine causal relationships due to the limitation on how child abuse was assessed. Finally, data collection ended in 2022; given the potential patterns of increased conflict identified in this study, and the risk of future pandemics, ongoing assessments of interpersonal relationships among children, youth and young adults with a child maltreatment history is needed to better understand the long-term impacts of such global emergencies.

Despite these limitations, knowledge generated through this study contributes to our understanding of the association between the COVID-19 pandemic for youth with and without a child maltreatment history, emphasizing the critical and ongoing need for support for families with histories of abuse, neglect, or violence (52, 68). Appropriate supports or interventions can buffer against risk factors and promote protective factors in the face of adversity (5, 16, 30, 69, 70); without these, interpersonal issues and relationship patterns experienced or established during childhood and adolescence may persist into adulthood, impacting health and well-being, quality of intimate relationships, future parenting styles, personal safety, and overall quality of life (5, 10, 50, 56, 67). Supports may include policies and programs implement at the individual level (e.g., adolescent intimate partner violence prevention programs) (58), at the family level (e.g., parent/caregiver supports and educational programs; policies that support families that are at economic disadvantages) (8, 46, 47), at the school level (e.g., safer school programs and policies, peer to peer supports, mental health services for students, school personnel training for signs and impacts of child maltreatment on student well-being) (5, 58), at the community level (e.g., community health workers trained to identify and support communities with high prevalence of violence) (1), and at the clinical level (e.g., enhanced clinician ability to recognize and enquire about child maltreatment and relational conflict in a safe environment, and respond appropriately if interventions are needed) (71). Given the inequities for youth with a child maltreatment history and potential ongoing challenges “post”-pandemic, it is timely and necessary to continue to progress our knowledge, address the long-term impacts of the pandemic, prevent worsening inequities, and strengthen youths' relationships with others to promote health and well-being now and into the future.

## Conclusion

The COVID-19 pandemic had significant impacts on youth health and well-being. Results from the present study contribute to our knowledge of the association between the COVID-19 pandemic and relational conflict among youth with a child maltreatment history. Youth with a child maltreatment history reported a greater likelihood of conflict with their parents, siblings, and intimate partners compared to youth without a child maltreatment history at the onset of the pandemic, and increased conflict remained significant in 2022 after most public health restrictions had ceased. The pandemic-related interpersonal challenges experienced by youth during a critical developmental life period may affect long-term biopsychosocial outcomes, leading to greater inequities for this population across the life course. Study results illustrated potential long-term challenges for youth with a child maltreatment history, compared to youth without child maltreatment experiences, and contribute to the knowledge of early and ongoing pandemic-related issues for this population. Ongoing research and supports for youth and families with maltreatment experiences is necessary at this point in history to prevent worsening outcomes and promote well-being for all.

## Data Availability

The data analyzed in this study is subject to the following licenses/restrictions: Data sharing is not permitted by the study ethics agreement. Requests to access these datasets should be directed to Tracie Afifi, tracie.afifi@umanitoba.ca.
